# Extraction and Characterization of New Cellulosic Fiber from *Catalpa bignonioides* Fruits for Potential Use in Sustainable Products

**DOI:** 10.3390/polym15010201

**Published:** 2022-12-30

**Authors:** Ebru Bozaci, Aylin Altınışık Tağaç

**Affiliations:** 1 Department of Textile Engineering, Ege University, Izmir 35030, Turkey; 2 Department of Chemistry, Dokuz Eylül University, Izmir 35390, Turkey

**Keywords:** *Catalpa bignonioides*, cellulosic fibers, sustainable

## Abstract

The purpose of this study was to investigate the extract of *Catalpa bignonioides* plants and characterize novel natural cellulosic fibers from the fruits as an alternative material for sustainable products. The *Catalpa bignonioides* tree contains pharmacologically active compounds and is found all over the world. The sustainable natural fibers were easily extracted in an environmentally friendly manner from the fruits of the plant and characterized in terms of their chemical, thermal, and physical properties. The *Catalpa bignonioides* fibers (CBF) were composed of cellulose (58.3%), hemicellulose (3.1%), and lignin (38.6%) and had a low density (0.713 g/cm^3^). Fourier transform (FT-IR) analysis, X-ray diffraction (XRD), and scanning electron microscopy (SEM) analyses were used to search for the chemical groups, crystalline structures, and surface morphology of the CBF fibers. The results suggest that CBF fibers are a suitable alternative for composite and textile applications.

## 1. Introduction

In the last few decades, increasing global warming, the depletion of resources, and environmental concerns have increased interest in sustainable production. These concerns have led researchers to search for new biodegradable natural materials to replace synthetic ones. Natural cellulosic fibers, which can be found in abundance in nature and can be easily extracted, have distinct properties such as low costs, low-level hazards, high strength-to-weight ratios, biodegradability, non-corrosiveness, good physical and mechanical properties, and easy processing [1,2,3,4,5]. Unconventional agricultural natural fiber sources, which are inexpensive, renewable, and sustainable resources created by nature, have become valuable alternative materials for commonly used cellulosic fibers such as cotton and linen since conventional fibers do not meet the increasing demand for natural fibers [6,7]. Cellulosic natural fibers can be extracted from the seeds, fruits, leaves, and bast of plants. They are mainly composed of cellulose, lignin, pectin, hemicellulose, and wax [8]. In recent years, quite a bit of research has been performed on the extraction of new natural fibers and the study of their properties [3,4,9,10,11,12,13,14,15].

*Catalpa bignonioides*, which belongs to the Bignoniaceae family, is native to southeastern North America and grown as an ornamental tree on streets and in gardens in almost all temperate climates around the world, and it is a medium-sized deciduous tree with a height of 15–18 m [16,17,18]. The Bignoniaceae family has been the subject of many studies due to pharmacologically active compounds such as steroids, tannins, etc. Studies have shown that these family members have laxative, sedative, antiseptic, anti-inflammatory, antidiuretic, antiarthritic, antimicrobial, antioxidant, and antitumoral properties [16,17,18,19,20,21]. The pods, seeds, leaves, and roots of *Catalpa bignonioides* have also been investigated for the treatment of ulcers, skin, respiratory diseases, scrofulous maladies, and helmintic infections [22]. Although there are many studies on the medical use of the *Catalpa bignonioides* plant, a study on the fibers obtained from its fruit could not be found in the literature. To the best of our knowledge, this is the first report on new cellulosic fiber from *C. bignonioides*. The main objective of this study was (1) to unearth whether fibers can be extracted from *C. bignonioides* fruits, (2) to provide chemical and physical data showing the properties of the fibers of *C. bignonioides* fruits, and (3) to provide important data for further studies on sustainable and cleaner products such as textile and composite applications. In this study, the fibers were extracted using the water-retting method on the fruits of *C. bignonioides*. The characteristics of the fibers were identified using chemical, physical, and thermal analyses such as Fourier transform infrared spectroscopy (FTIR), tensile test, scanning electron microscopy (SEM), density, chemical composition, X-ray diffraction (XRD), and thermogravimetric analysis (TGA). Consequently, the results showed that fibers extracted from *Catalpa bignonioides* fruits have potential as textile fibers.

## 2. Materials and Methods

### 2.1. Materials

Cellulose fibers can be released from the surrounding tissues of plants using the retting process. This can be performed either with microorganisms penetrating the plant body or enzymatic processes by converting pectinase, which binds the fiber bundles together into simple water-soluble compounds. Retting can be performed in dew, still water, running water, and hot water and chemically, mechanically, or enzymatically. Water-retting is based on fermentation with anaerobic bacteria, yielding a high fiber quality [23,24].

**Figure 1:**
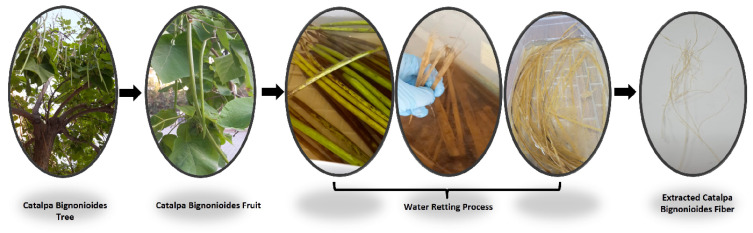
*Catalpa bignonioides* tree and fruit.

*Catalpa bignonioides* fibers (CBFs) were isolated from the plants’ fruits and harvested in the summer via the water-retting process, and the morphology of the fibers was characterized (Figure 1). The extracted fibers were washed 5 times with tap water after the retting process to remove any impurities and then dried in an ambient temperature. NaOH, H_2_SO_4_, NH_3_, and HCl were purchased from Sigma-Aldrich [24,25,26].

### 2.2. Methods

A chemical composition analysis was performed to determine the different chemical components of the fibers (cellulose, hemicellulose, and lignin). Before the chemical composition analysis, the fibers were subjected to a moisture removal process at 105 °C for 4  h in an oven. The detailed steps of the analyses are provided elsewhere [27,28,29]. Fiber morphology was analyzed with scanning electron microscopy (SEM). The micrographs were carried out using a COXEM EM-30 Plus scanning electron microscope at different magnifications. The FT-IR spectra of the samples were determined using a Perkin Elmer (Spectrum 100,Waltham, MA, USA) instrument in the attenuated total reflectance (ATR) mode using a diamond/zinc selenide crystal at 4000–6500 cm^−1^. The lateral order index (LOI) of the CBF fiber was calculated using the spectral ratio of the absorbencies (1421/894) cm^−1^ [29,30]. X-ray diffraction (XRD) analyses were carried out at a scan rate of 0.02 s^−1^ with a diffraction angle range of 2θ = 3–40° (Thermo Scientific ARL X’TRA, Ecublens, Switzerland). The thermograms (25 to 600 °C, 5 °C/min heating rate, under nitrogen atmosphere) were taken with a thermogravimetry/differential thermal analyzer (PerkinElmer Diamond, Waltham, MA, USA). The crystallinity index (CI %) of the fibers and the average size of the crystals were calculated using the Segal empirical method and Scherrer’s equation, described, respectively, in [26,29]. X-ray photoelectron spectroscopy (XPS) was used to identify the surface chemistry and characteristics of the fibers. Measurements were taken between 1350 and 10 eV at 1 eV resolution using a Thermo Scientific device and an Al-Kα X-ray source (1486.7 eV). The sample surface was cleaned with inert gas before the analysis and recorded with 10 scans. Fiber density was measured with a standard pycnometer according to the mass difference technique (ASTM D2320-98-2003). The tensile strength was determined according to ASTM D3822-01 [11,29].

## 3. Results and Discussions

### 3.1. Chemical Composition

Cellulose, hemicellulose, and lignin, as the main constituents of CBF fibers, accounted for 58.3%, 3.1%, and 38.6%, respectively. Although the lignin content of the CBF fiber was similar to coir fiber (32.69%), the cellulose content (42.10%) was higher, and the hemicellulose content (22.56%) was lower [30]. The cellulose ratio of CBF fiber is comparable, but the lignin ratio is higher than in some bast fibers [29]. The degradation rate of lignin at high temperatures is lower than that of cellulose and hemicellulose; therefore, its resistance to high temperatures is higher than that of cellulose and hemicellulose. The high lignin content of the CBF fiber is thought to provide good thermal stability [31,32,33,34,35].

### 3.2. Density

Lightweight materials are important for the aerospace, marine, textile, and automobile industries. Accordingly, the extraction and characterization of new, low-density, biodegradable, sustainable, and environmentally friendly natural fibers for industrial applications are of great importance [34]. The density of the *Catalpa bignonioides* fruit fiber was found to be 0.713 g/cm^3^, which is lower than coir and most of the common bast fibers, which are used in polymer composite applications. As natural fibers possess less density than synthetic fibers, *Catalpa bignonioides* fiber can also be a good alternative to lightweight polymer composites [36,37]. Natural hollow fibers showing low specific density are very limited, and CBF fiber can also be a good candidate to replace kapok fibers in terms of having a higher surface area and better tensile strength [38].

### 3.3. Scanning Electron Microscopy (SEM) Analysis

**Figure 2:**
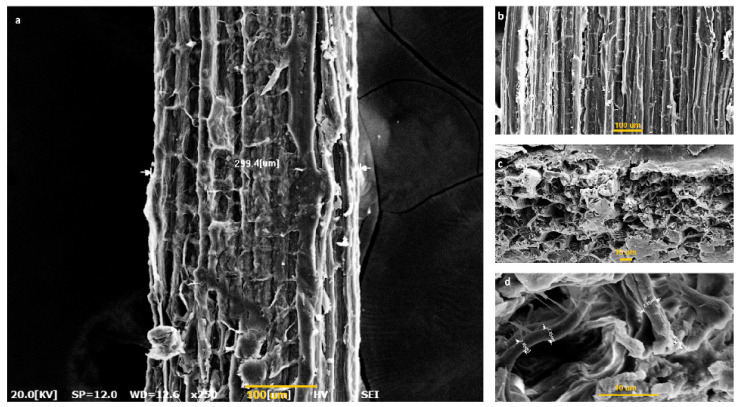
SEM images of *Catalpa bignonioides* fiber: (**a**,**b**) longitudinal view of the fiber (×250); (**c**,**d**) cross-section of the fiber (×1.0k and ×5.0k).

Figure 2a shows that there are many indentations and protrusions on the fiber surface formed by microfibrils, increasing the fiber surface roughness and micropores. It is clear from Figure 2b that microfibrils are formed uniformly along the fiber. This placement is believed to result in the high strength of the fibers. Micropores and the high roughness of the fiber surface increase the interfacial adhesion between the fiber and matrix when used in composites. This also causes an increase in the surface area, thus improving the spinning and dyeing properties of the fiber [26,37,39]. Figure 2c,d illustrate the cross-section SEM micrographs of the *Catalpa bignonioides* fibers. The cross-section micrographs of the fiber demonstrate that it has a round and hollow section and contains different layers, such as hemicellulose, pectin, and fiber cellulose. The hollow structure gives the fiber a low specific density and the ability to hold air and water vapor. It is known that hollow structure fibers can be used not only in composites but also in clothing, heat insulation, sound absorption, oil absorption, buoyancy, and bulk materials [26,38]. Each fiber bundle consists of several single fibers ranging in width from 1.9 μm to 2.9 μm. The average width of the fibers obtained from the fruit of the plant was found to be 299 μm, and the length was 20–35 cm. The fiber fibrils were composed of microfibrils brought to the median by the elementary fibrils [23].

It was determined from the longitudinal fiber section in Figure 2a,b that the *Catalpa bignonioides* fiber sample had a diameter of about 299 µm. The hollow structure of the fiber (Figure 2c,d) gives it a bulky structure and low density, which provides an advantage for its use in composite structures. It can be clearly seen that the catalpa fiber was composed of many microfibers linked together by trace amounts of non-cellulosic compounds [40,41].

### 3.4. Fourier Transform Infrared Spectroscopy (FTIR-ATR) Analysis

**Figure 3:**
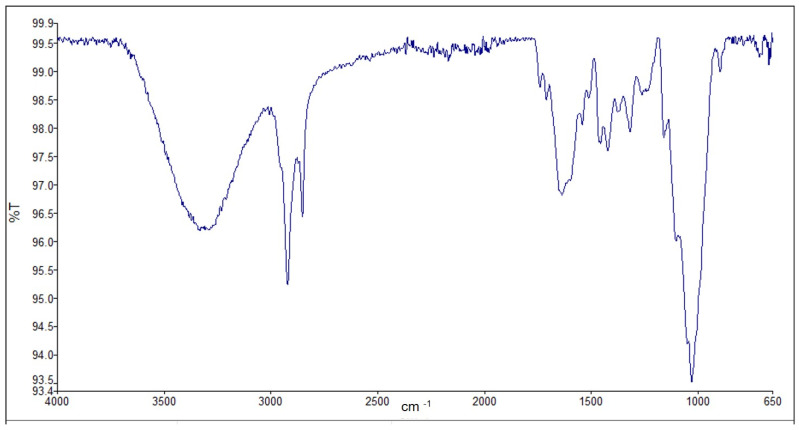
FTIR spectrum of *Catalpa bignonioides* fiber.

Natural cellulose-based fibers are mainly composed of cellulose, hemicellulose, lignin, and wax, which contain functional groups such as alcohol, ketone, ester, alkenes, and benzene. As shown in the FTIR-ATR spectra of the *Catalpa bignonioides* fibers in Figure 3, the peaks observed at 3331 cm^−1^ and 2919 cm^−1^ are due to the stretching vibration of OH and inter- and intramolecular vibrations in CH bonds in polysaccharides. The bands at 3331 cm^−1^, 1262 cm^−1^, and 1025 cm^−1^ are related to the cellulose components of the fiber. The band at 2918 cm^−1^ and a weak shoulder at about 2800 cm^−1^ are ascribed to a C-H stretching vibration of hydrocarbon constituents (cellulose and hemicelluloses) in polysaccharides. The weak shoulder is probably caused by the low hemicellulose content of *Catalpa bignonioides* fibers. The band at 1597 cm^−1^ may be associated with water molecules absorbed by the fiber or the antisymmetric stretching of COO−. The C=C stretch of the aromatic ring and the C-H out-of-plane vibration of the lignin are represented by the peaks at 1500 cm^−1^ and 833 cm^−1^, respectively. The absorption bands located at 1421, 1372, 1319, 1025 cm^−1^, and 894 cm^−1^ belong to CH_2_ symmetric bending, C-H bending, OH deformation bands, the antisymmetric deformation of the C-O-C band, and the C-H out-of-plane vibration in cellulose, respectively. The band at 1421 cm^−1^ provides information about the crystalline amount of cellulose, and the band at 894 cm^−1^ provides information about the amorphous region [42,43,44]. The peaks at 1421 cm^−1^ and 895 cm^−1^ were used to determine the nature of the crystallinity of the CBF fibers. The lateral order index (LOI) indicates the order of crystallinity relative to the amorphous constituents rather than the amount of crystalline cellulose [29,45]. According to Manjula et al. 2017, the LOI value of raw coir can be calculated as 0.0835, and after NaOH treatment, the value can increase up to 0.429. The value of raw CBF was calculated as 0.97; this may be due to the lower amorphous cellulose content [30].

### 3.5. X-ray Diffraction (XRD) Analysis

**Figure 4:**
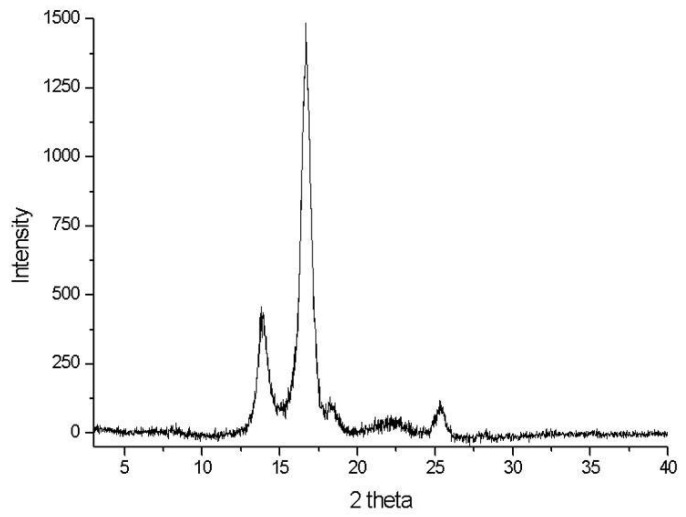
XRD pattern of *Catalpa bignonioides* fiber.

The crystalline nature of fibers can be determined using the X-ray diffraction (XRD) method [30]. The degree of crystallinity in the *Catalpa bignonioides* fibers was analyzed using an XRD pattern (Figure 4)

As is seen in Figure 4, the major crystalline peak of the *Catalpa bignonioides* fibers appears at 2θ = 16.6°. The *Catalpa bignonioides* fibers have diffraction peaks at 13.8°, 16.6°, and 22.2°, corresponding to the (101), (101), and (002) planes, respectively, and these diffraction peaks can be applied to the characteristic diffractions of cellulose I. The crystallinity index of *Catalpa bignonioides* is 94% (Figure 4). The calculated CI value is considerably higher than the other commonly used natural fruit cellulose fibers (Coir 50.8%). The CI value is considered to be high due to the low content of non-cellulosic substances [29,30].

### 3.6. XPS

**Figure 5:**
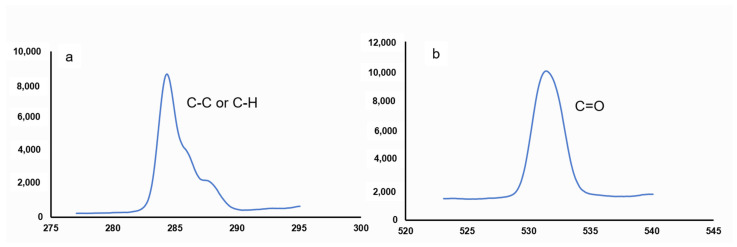
High-resolution XPS spectra of (**a**) C1s and (**b**) O1s peaks.

X-photo electron spectroscopy (XPS) analysis is a useful method of assessing the surface chemistry of fibers and determining the elemental distribution and quantity of elements (C, O, N, Si, etc.) in terms of weight (%) [46,47]. The weight percentage and ratios of the prominent elements distributed on the surface of the *Catalpa bignonioides* fibers and C1s and O1s peak spectra are provided in Table 1 and Figure 5, respectively [48].

Carbon (49.87%) and oxygen (31.35%) are the main elements of the surface of *Catalpa bignonioides* fibers. This may be due to the high cellulose content, as demonstrated by our crystallinity analysis. Cellulosic fibers are mainly composed of carbon and oxygen in varying proportions, and *Catalpa bignonioides* fibers contain a similar amount of carbon as commercial fibers such as cotton (46.1%), flax (55.68%), and jute (62.65%) do [48]. The deposition of phosphorus, sulfur, calcium, nitrogen, sodium, silicon, and magnesium elements on the surface of the fibers is probably due to impurities in the fiber surface [48,49].

The C/O and O/C ratios for *Catalpa bignonioides* fibers were calculated as 1.59 and 0.63, respectively. These ratios can be used to determine the surface hydrophobic characteristics of the fibers, which are an important indicator of the compatibility of the fiber/matrix in composite production. As a result, the higher C/O in the *Catalpa bignonioides* fibers—indicating the hydrophobic surface properties—makes them suitable for environmentally friendly composite production [41,50].

The O/C ratio of the *Catalpa bignonioides* fibers was found to be higher than some known commercial fruit fibers such as cotton (0.14) and coir (0.29) and bast fibers such as flax (0.14), jute (0.46), and kenaf (0.45) [27,28,51,52,53]. The higher O/C ratio is probably due to the presence of non-cellulose components such as hemicellulose, lignin, and waxes on the fiber surface in lower amounts [41].

The peaks located at 285.35 and 532.21 eV are associated with the C–C/C–H and O = C groups, respectively [35,41,50,54,55]. The XPS peaks are consistent with the FTIR results.

### 3.7. Thermogravimetric (TGA) Analysis

**Figure 6:**
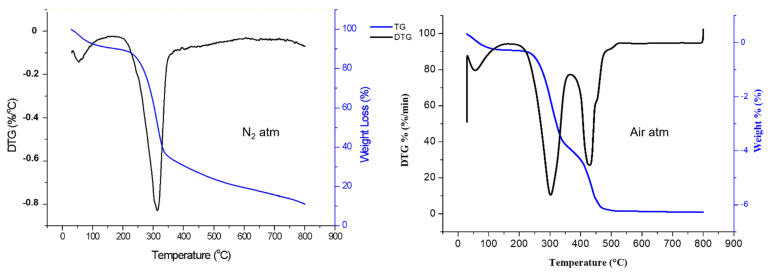
Thermogravimetric analysis of *Catalpa bignonioides* fiber (N_2_ and air atmospheres).

The thermal decomposition behavior of *Catalpa bignonioides* fibers was studied using TG and DTG curves (Figure 6), and the results are summarized in Table 2.

Fiber-reinforced polymer composites can be produced at relatively high temperatures [30,41]. Therefore, the thermal stability of cellulosic fiber is one of the most important parameters for composite production. The thermal degradation of CBF occurred in two phases; initial degradation was probably due to the removal of water and wax (56 °C and 10%). The second phase of thermal degradation occurred at 244.21 °C due to the degradation of hemicellulose, with a very small amount of weight loss. The third degradation peak occurred at 314 °C with a weight loss of 71%, indicating the deterioration of α-cellulose. Similar peaks have been reported for various cellulosic fibers such as bamboo, hemp, jute, and kenaf at 321 °C, 308 °C, 298 °C, and 307 °C, respectively [44,56,57]. The region between 500 °C to 650 °C indicated the degradation of the charred residue [13,15,52,58,59]. The thermal degradation of CBF in an air atmosphere was also studied. Although the degradation temperature (Td) was defined as the temperature at a 5 wt% weight loss, the temperature at the maximum weight loss rate (Tmax) was found to be similar in both the N_2_ and air atmosphere conditions. The maximum decomposition rate (Rmax) was higher in the air atmosphere than in the N_2_ atmosphere, so char yields were found to be significantly different, as expected [60].

### 3.8. Tensile Strength

The tensile strength and elongation at the break of the *Catalpa bignonioides* fiber were measured as 691.1 ± 55.8 MPa and 4.24% ± 0.9, respectively. Although *Catalpa bignonioides* fiber is a fruit fiber, the tensile strength value was found to be similar to many bast fibers, such as hemp, flax, and ramie. The new *Catalpa bignonioides* fiber is better in terms of tensile strength than coir (175 MPa), one of the most common commercial fruit fibers [29,61,62]. The thermal and mechanical properties of the fibers are mainly affected by elemental content. The high cellulose and low hemicellulose content of the CBF probably enhance the tensile strength of the fiber [34].

## 4. Conclusions

In this study, a new cellulose fiber was successfully extracted from *Catalpa bignonioides* tree fruits using the water-retting process. The chemical, morphological, and thermal properties of *Catalpa bignonioides* were thoroughly investigated. The cellulose, hemicellulose, and lignin contents of the fibers were found to be 58.3%, 3.1%, and 38.6%, respectively. Chemical analyses, FT-IR, and XRD examinations showed the existence of cellulose and non-cellulosic materials in the fiber. Thermogravimetric analysis of fibers showed a thermal degradation at 314 °C, similar to other cellulosic fibers. SEM images showed the hollow structure of the fiber; together with their low-density value, this suggests alternative uses for CBF fibers, not only as composites but also in clothing, heat insulation, sound absorption, oil absorption, buoyancy, and bulk materials. From these findings, it can be concluded that *Catalpa bignonioides* fibers might be a possible alternative as sustainable and biobased materials for many industries, such as in composites and textiles.

## Figures and Tables

**Table 1:** The weight percentages and ratios of the surface element compositions of the *Catalpa bignonioides* fibe.rs.

Element Survey	C1s (%)	O1s (%)	Ca2p (%)	P2p (%)	N1s (%)	S2p (%)	Mg1s (%)	Si2p (%)	Na1s (%)	C/O	O/C
	49.87	31.35	8.21	3.53	2.11	1.72	1.24	1.03	0.95	1.59	0.63

**Table 2:** TGA data in air and nitrogen atmospheres.

Td °C		Tmax °C		Rmax %/min		CY(Air)-800 °C	CY(N2)-800 °C
(Air)	(N2)	(Air)	(N2)	(Air)	(N2)		
67	68	301	314	5.760	4.120	0.93	11

## Data Availability

The data presented in this study are available upon request from the corresponding author.
